# Mechanics of spreading cells probed by atomic force microscopy

**DOI:** 10.1098/rsob.130084

**Published:** 2013-07

**Authors:** Anna Pietuch, Andreas Janshoff

**Affiliations:** Institute of Physical Chemistry, Georg-August-University of Göttingen, Tammannstrasse 6, 37077 Göttingen, Germany

**Keywords:** membrane tension, cell mechanics, adhesion, spreading

## Abstract

Cellular adhesion and motility are fundamental processes in biological systems such as morphogenesis and tissue homeostasis. During these processes, cells heavily rely on the ability to deform and supply plasma membrane from pre-existing membrane reservoirs, allowing the cell to cope with substantial morphological changes. While morphological changes during single cell adhesion and spreading are well characterized, the accompanying alterations in cellular mechanics are scarcely addressed. Using the atomic force microscope, we measured changes in cortical and plasma membrane mechanics during the transition from early adhesion to a fully spread cell. During the initial adhesion step, we found that tremendous changes occur in cortical and membrane tension as well as in membrane area. Monitoring the spreading progress by means of force measurements over 2.5 h reveals that cortical and membrane tension become constant at the expense of excess membrane area. This was confirmed by fluorescence microscopy, which shows a rougher plasma membrane of cells in suspension compared with spread ones, allowing the cell to draw excess membrane from reservoirs such as invaginations or protrusions while attaching to the substrate and forming a first contact zone. Concretely, we found that cell spreading is initiated by a transient drop in tension, which is compensated by a decrease in excess area. Finally, all mechanical parameters become almost constant although morphological changes continue. Our study shows how a single cell responds to alterations in membrane tension by adjusting its overall membrane area. Interference with cytoskeletal integrity, membrane tension and excess surface area by administration of corresponding small molecular inhibitors leads to perturbations of the spreading process.

## 2. Introduction

Cellular adhesion and motility are fundamental for biological processes such as morphogenesis, wound healing and tissue homeostasis. For instance, lymphocytes migrating through an intact cellular layer rely on the ability to adhere and move through tissue [1]. Sahai & Marshall [2] report that tumour melanoma cells produce dynamic blebs, which allow migration through a three-dimensional matrix. Experience from these studies clearly shows that cellular deformation and supply of excess membrane are key factors for the above-mentioned processes. While the morphological changes taking place during spreading of cells to a substrate or matrix are well known and characterized for many different cell lines, comprehensive studies of the accompanying changes in cell mechanics are scarce, although it is known that membrane tension and cell motility are strongly interwoven [3]. Generally, the spreading process can be divided into three phases [4,5]. The first phase is characterized by the formation of initial bonds between adhesion molecules and molecules of the extracellular matrix (ECM). This is followed by the second phase, comprising the initial cell spreading, which is driven by actin polymerization that forces the cell surface area to increase by drawing membrane from a reservoir of folded regions and blebs. Blebs are characteristics not only of apoptosis [6] and cell division [7], but also for cell spreading [8]. Cells in suspension frequently display blebs, which originate from a drop in tension due to local disassembly of the actin cortex. The same is formed upon detachment of an adhered cell. These membrane protrusions appear transiently only during the spreading progress and vanish at a later stage [8]. After the depletion of this membrane reservoir, the cells enter the third spreading phase, whereby additional plasma membrane is recruited from internally stored membrane buffer (i.e. excess area) [9]. The various stages are accompanied by substantial changes in cellular mechanics to accommodate the mechanical challenges posed by spreading and migration. In particular, volume changes pose a substantial threat to membrane integrity due to the lateral inextensibility of the membrane, which does not allow for area dilation beyond a few per cent [10].

Here, we study the mechanics of cell spreading on a surface as a paradigm for the close relationship between cell shape and membrane mechanics. Spreading of a cell is an F-actin-dependent process as cells spread very slowly after disruption of the actin cytoskeleton [11], whereas the plasma membrane undergoes a mechanical deformation from a spherical shape to a nearly discoidal morphology promoting the idea that membrane mechanics (i.e. the membrane's in-plane tension), participate in cell spreading regulation. Stretching an epithelial cell causes the cell to sacrifice lateral protrusions, whereas suppression of spreading activity is reversed upon release of tension [12]. Raucher & Sheetz [13] reported that chemically induced reduction of membrane tension leads to faster lamellipodial extension. This suggests that the plasma membrane acts as a regulator of cell spreading through tension homeostasis, limiting the number of protrusions owing to exhaustion of membrane reservoirs. Indentation experiments combined with site-specific pulling of membrane tethers, both carried out with the atomic force microscope (AFM) at the same spot, allowed us to assess simultaneously local changes in membrane tension, cortical tension *and* excess area during the spreading of epithelial cells. During adhesion and spreading, the shape of a suspended cell changes from a sphere to a capped sphere. Cell shape changes were taken into account by adjusting the geometrical constraints of a tension model describing the cell as a liquid-filled body with an isotropic in-plane tension [14,15]. These experiments are supported by fluorescence microscopy of the plasma membrane as well as visualization of F-actin of adhering and spreading cells. Administration of toxins targeting cytoskeletal integrities, membrane tension and surface area availability confirms that the process of spreading is not very robust and is easily perturbed. We were able to show that the adhesion and spreading leads to a remarkable and dynamic change in the mechanical properties of the cell, and that cells regulate and restore changes in membrane tension during adhesion and spreading by adapting their excess surface area accordingly.

## 3. Results and discussion

### 3.1. Changes in the cellular mechanics during cell spreading: model-free characterization

**Figure 1.**
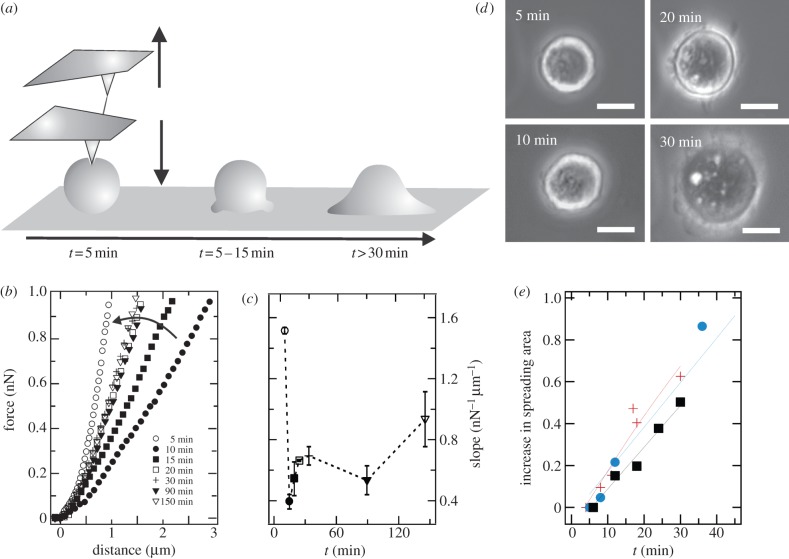
Mechanical properties of MDCK II cell during adhesion and spreading. (*a*) Scheme illustrating the adhesion and spreading process. The adhering and spreading cell is locally indented with an AFM tip, resulting in force curves shown exemplarily in (*b*). While retracting the cantilever, a membrane nanotube (tether) is frequently pulled out of the plasma membrane, bearing additional mechanical information (equation (3.7)). (*b*) Representative force curves taken at different time points during cell spreading. The force curve recorded 5 min after cell seeding (open circles) shows the steepest slope, followed by a sudden softening (10 min, black circles). Within 30 min, force curves recover to higher stiffness. (*c*) Slope of FCs obtained from large strains plotted against time. (*d*) Light microscopy images taken at different time points documenting the spreading behaviour of MDCK II cells. Scale bar, 10 µm. (*e*) Increase in projected and normalized spreading area ((*A*_sp −_ *A*_sp_(0)/*A*_sp_(0)) over time from three individual MDCK II cells.

We monitored the mechanical behaviour of a spreading MDCK II cell as a function of time, using force measurements to characterize this process in terms of varying membrane tension and concomitant surface area regulation (figure 1*a*). Indentation of a cell with an AFM tip provides force versus tip–sample-separation curves (FC). Generally, a larger slope corresponds to a stiffer cell. Figure 1*b*,*c* shows a shift in the slope of FCs taken on a single cell during adhesion and spreading, which is generally observed in all experiments (3/3). The first FC is taken 5 min after cell seeding on the centre of the cell body, with an indentation depth of 1 µm to reach the given set point of 1 nN normal force. Five minutes later, we observe a tremendous decrease in the slope of the FC corresponding to a considerable softening of the cell, mirrored in an increased indentation depth of 3 µm. Following the spreading process over the next 3 h reveals a reversal of the slope towards its initial value (i.e. the cell body becomes stiffer again; figure 1*b,c*). Even without an appropriate mechanical model of the cell, a change in the slope is indicative of a substantial but also reversible change in the cell's stiffness during spreading. A higher indentation depth at constant force could be reached only if the cells soften, or, more precisely, if the cells obtain access to a membrane reservoir. The latter would allow compensation for the stress exerted by the indenter. This initial softening is followed by a gradual increase in cell stiffness towards a constant value (more than 20 min). Simultaneously, we record optical phase contrast images to concomitantly follow cell shape changes during adhesion and spreading (figure 1*d*). Figure 1*e* shows the time course of the spreading area obtained from data analysis of the optical micrographs. Within the first 40 min, the contact area increases linearly.

**Figure 2.**
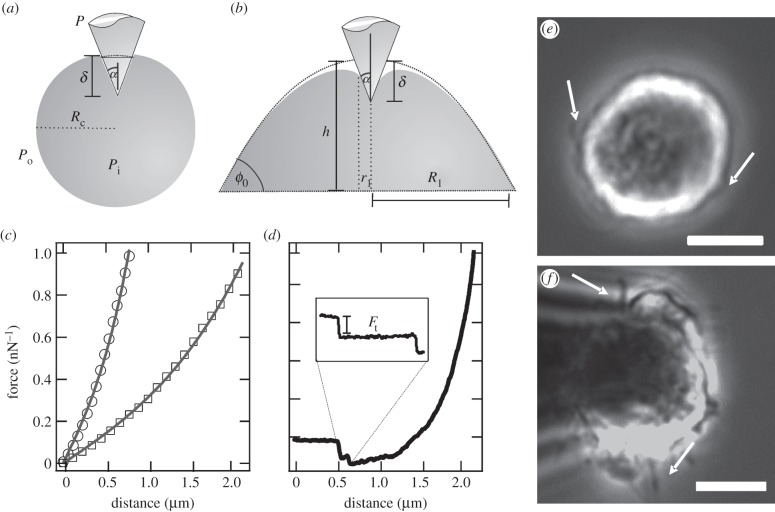
Mechanical model of adhering and spreading MDCK II cells. (*a*) Illustration of a conical probe indenting a spherical, non-adhering (left) and spreading (right) cell. *P*_i_, *P*_o_ and *P* are the pressure of the cell interior, fluid surrounding the cell and the external pressure exerted by the tip. The opening angle of the indenter cone is *α*. *R*_c_ is the radius of the cell. (*b*) The spreading cell (more than 15 min) is described by a spherical cap [15]. *R*_1_ is the base radius, *r*_1_ the radial position beyond which the membrane is not in contact with the tip, *ϕ*_0_ is the contact angle between cell and substrate, *δ* is the indentation depth and *h* is the cell height before indentation. (*c*) Force curves taken on a spherical cell (circles) and spreading cell after 15 min (squares) with the corresponding fit using membrane theory (see text). (*d*) Retrace curve displays tether formation. (*e*) Phase contrast micrograph of a suspended MDCK II cell. Cells display small blebs (arrows). (*f*) Phase contrast micrograph of a suspended MDCK II cell treated with cytochalasin D to disrupt the cortex. These cells display abnormal membrane outgrowths (arrow). Scale bar, 10 µm.

Cells in suspension exhibit a spherical shape, frequently displaying blebs (figure 1*d*, 5 min; figure 2*e*, arrows). Moreover, when suspended cells are treated with F-actin-destabilizing cytochalasin D, cells show a considerable increase in the membrane outgrowths (figure 2*f*). First, significant cell shape changes can be observed after 10 min, where spherical membrane protrusions become visible, forming the initial substrate adhesion area. This initial adhesion phase is followed by a substantial increase in adhesion area (20–30 min), which we refer to as the initial spreading phase. The dynamics of cell spreading has been studied for many cell lines, drawing a comprehensive picture of this process. Bereiter-Hahn and co-workers [16] showed that the initial phase is characterized by the attachment of the cell to the substrate, forming a broad and smooth contact area (within the first 10 min) followed by a broadening of the adhesion zone through cytoplasmic flow. The final phase in cell spreading is characterized by the extension of lamellae [16]. Observing the spreading area over time, Norman *et al*. [8] found that the contact area remains constant after 40–60 min. We observe a similar time-dependent spreading behaviour for MDCK II cells, leading to the chronological sequence illustrated in figure 1*a*. The next step in describing the mechanical response is to devise an appropriate model of the cell that not only captures the essential mechanical features encompassing tension, area compressibility and surface area, but also takes into account morphological changes. The cell shape needs to be considered carefully as it bears important input parameters for any mechanical model. Otherwise, changes in cell shape might falsely be attributed to changes in the mechanical parameters [17].

### 3.2. Mechanical model

The mechanical model used to describe force indentation experiments of cells in the context of a single spreading cell relies on the assumption that the restoring force in response to indentation originates mainly from a constant isotropic tension *T*, comprising the sum of cortical and membrane tension *T*_0_ and a term describing the stretching or area dilatation of the bilayer [15]:3.1
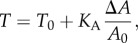
where *ΔA* denotes the change in surface area related to the initial surface area of the cell *A*_0_ and *K*_A_ denotes the area compressibility modulus of the plasma membrane in conjunction with the cortex. Because the area compressibility modulus *K*_A_ of a lipid bilayer is usually in the range of 0.1–1 N m^−1^, depending on the lipid composition in comparison with the rather large compliance of the actin cortex (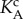 approx. 0.001 N m^−1^), bilayer mechanics and resistance to stretching dominate the response to large strains [18]. Concretely, assuming a typical thickness of the cortex in the range of 1 µm and a Young's modulus of 1 kPa, we arrive at a 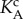 of about 0.001 N m^−1^, which is more than two orders of magnitude lower than the *K*_A_ of a fluid lipid bilayer. The large value for *K*_A_ is due to the liquid–crystalline nature of the bilayer giving rise to a lateral inextensibility (i.e. the bilayer already ruptures at *ΔA*/*A*_0_ ∼ 2–3%). At small strains, however, the overall tension *T*_0_ dominates the mechanical response. *T*_0_ comprises the active cortical tension *T*_c_, [19,20] generated by active contraction of the actomyosin cortex, and the membrane tension *T*_t_ that encompasses membrane's in-plane tension *γ*_m_ and the tension resulting from cytoskeleton–membrane attachment sites *γ*_ad_:3.2
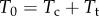
and3.3
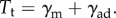
The real surface area of the cell is usually considerably larger than the geometrical one (*A*_0_) due to wrinkles and folds of the membrane on smaller length scales. Therefore, the area compressibility modulus needs to be replaced by the so-called apparent area compressibility modulus 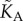:3.4
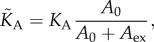
where 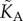 is usually smaller than *K*_A_ due to the presence of excess membrane area leading to an apparent reduction of *K*_A_. Therefore, smaller *K*_A_ values correspond to a large surface excess. In order to describe the mechanics of the cells with this tension model, it is important to compute the correct shape of cells during indentation. Here, we choose for the initial spreading phase a model described by Rosenbluth *et al*. [14] where the cell is treated as an unperturbed sphere (radius *R*_c_) prior to indentation (figure 2*a*). While the initial model assumes a pyramidal indenter, we slightly modified the theory to describe indentation with a conical tip. The force experienced by a conical indenter amounts to3.5
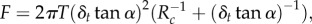
with3.6
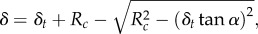
where *T* denotes the isotropic lateral tension, *δ*_*t*_ is the height of the cone immersed in the cell with an opening angle *α* and *δ* denotes the overall indentation depth (see electronic supplementary material, figure S1).

Notably, *T* is also a function of the indentation depth because it depends on the area dilatation (equation (3.1)). Fifteen minutes after adhesion, we switch to a model first described by Discher and co-workers [15], who compute the exact cell shape starting from a spherical cap (figure 2*b*). The cell shape is obtained from the radius of the contact zone *R_i_* and the wetting angle *ϕ*, as illustrated in figure 2*b* assuming constant curvature at a given indentation depth and constant volume as boundary conditions [21].

Cell indentation experiments are accompanied and verified by tether-pulling experiments carried out on the same spot. Tether pulling represents an independent mechanical approach for probing membrane properties. Upon retraction of the tip, adhesion permits a formation of a membrane nanotube containing only cytoplasma. Membrane tension contains contributions from membrane–cytoskeleton attachment *γ*_ad_, the contraction of the actomyosin cortex as well as tension in the bilayer itself *γ*_m_ [22]. Tether formation is characterized in FC (retraction) by a force plateau followed by a sudden release of force to zero owing to tether rupture, if the membrane reservoir is exhausted. This drop in force is called the tether force *F*_t_ (figure 2*d*). During tether formation, additional membrane material from pre-existing reservoirs is flowing into the growing tether. The force on the tether is directly connected to membrane tension *T*_t_ and bending rigidity *κ* of the membrane [17–20]:3.7
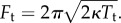
Therefore, we functionalized AFM tips with concanavalin A, providing enough adhesion to extract membrane nanotubes more frequently from the apical membrane than non-specific interaction would allow (figure 1*a*). In summary, the extraction of tethers allows us to determine the membrane tension separately from indentation experiments. Together with equation (3.2), we obtain access to *T*_c_ and *T*_t_, as well as 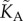 mirroring the excess surface area.

### 3.3. Mechanical parameters during adhesion and spreading

**Figure 3.**
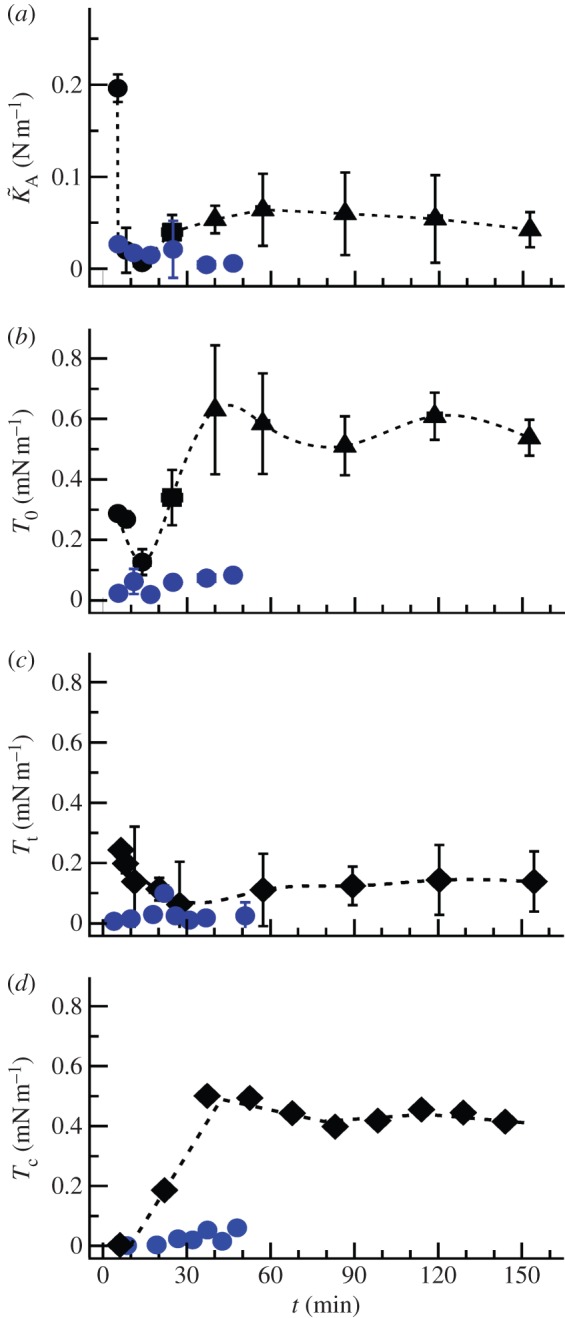
Mechanical parameters obtained from fitting force cycles taken during adhesion and spreading of untreated and cytochalasin D-treated MDCK II cells. (*a*) Apparent area compressibility modulus. (*b*) Membrane tension *T*_0_ obtained from indentation experiments. (*c*) Membrane tension *T*_t_ calculated from tether-pulling experiments assuming a bending module *κ* of 2.7 × 10^−19^ J. (*d*) Cortical tension calculated using equation (3.2). Black symbols indicate untreated cells, whereas blue symbols represent cytochalasin D-treated ones. Circles correspond to model shown in figure 2*a*, whereas squares represent mechanical parameters obtained from model shown in figure 2*b* with *ϕ*_0_ = 75°. Triangles represent modelling of a spread cell with *ϕ*_0_ = 21°.

The mechanical parameters obtained from shape-dependent FC fitting display a marked change in the initial phase of adhesion and spreading (figure 3*a–d*). The apparent area compressibility modulus 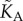, reflecting the change in membrane area, drops from (0.19±0.01) N m^−1^ (5 min) to a value of (0.006±0.006) N m^−1^ (15 min), indicative of a substantial increase in available membrane area. This is followed by an increase in 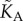, which levels off to an almost constant modulus of (0.05±0.02) N m^−1^. At the same time, we detect a decrease in the membrane tension *T*_0_ from an initial value of (0.29±0.06) mN m^−1^ to (0.13±0.04) mN m^−1^ (figure 3*b*). After this relaxation (15 min), the tension recovers to an almost constant value of (0.6±0.05) mN m^−1^.

The tension *T*_t_ determined from tether pulling also decreases within the first 30 min of adhesion and spreading from (0.19±0.01) to (0.06±0.05) mN m^−1^. After this initial drop, *T*_t_ eventually increases to a final value of (0.14±0.04) mN m^−1^. Both mechanical measurements (indentation and tether pulling) reveal the same trend in tension—first a decrease, followed by an increase, and eventually a constant value. Using equation (3.2) allows us to compute cortical tension from *T*_0_ and *T*_t_. Interestingly, we found that *T*_c_ is initially close to zero but increases within 30 min to reach a plateau (*T*_c_ ∼ 0.44±0.04 mN m^−1^). This is in accordance with findings of Arthur & Burridge [23], who also report a first loss of contraction between 10 and 30 min after seeding, to allow for spreading followed by a recovery of contractility. After 40–50 min, cortical tension dominates the mechanical response to small strains. Further spreading and polarization of a single cell (24 h) does not change tension any more. Finally, we obtain from indentation experiments a *T*_0_ value of 0.6 mN m^−1^ and from tether pulling a *T*_t_ value of 0.14 mN m^−1^.

### 3.4. Spherical cell: initial adhesion prior to spreading

MDCK II cells in suspension frequently display membrane protrusions that are lined with actin filaments (see electronic supplementary material, figure S2). Therefore, size and growth dynamics depend on cortical tension [20]. A sudden drop in tension, as it occurs upon detachment of an adherent cell, nucleates formation of protrusions, probably due to the contractile nature of the actomyosin cortex that restores tension in the plasma membrane through increase in internal pressure. The overall tension of the plasma membrane is a result of contributions from in-plane tension of the lipid bilayer, molecular adhesion of the plasma membrane to the underlying actin–cytoskeleton [24] and an active contribution from the contractile actomyosin cortex [25,26]. When membrane tension is chemically decreased, faster lamellipodial extension in spreading cells is observed, supporting the idea that cell membrane mechanics play an important role in controlling membrane protrusions, and therefore spreading [13].

**Figure 4.**
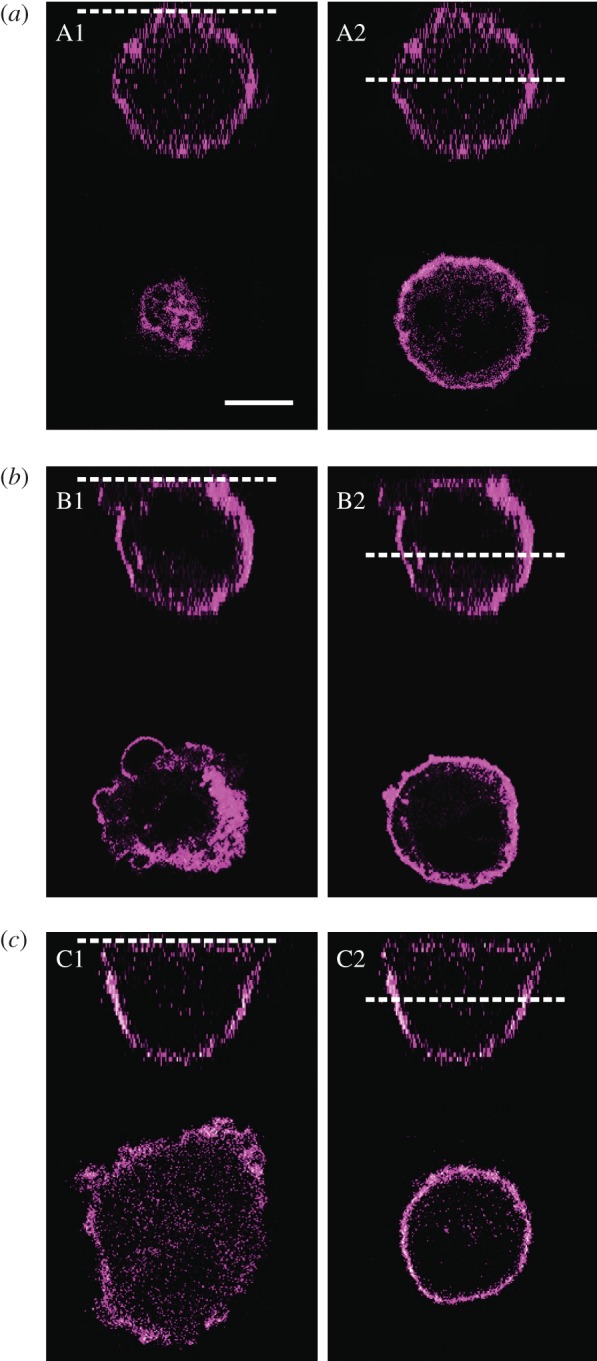
CLSM-fluorescence micrographs of the plasma membrane of MDCK II cells. (*a*) Fluorescence micrographs of a spherical cell 5 min after cell seeding. A1 shows the lower part of the cell, whereas A2 displays the middle plane of the cell. (*b*) Adhering cell 15 min after cell seeding. B1 and B2 show the basal section and the centre cross section, respectively. (*c*) Spread cell 30 min after seeding. C1 and C2 correspond to the section cuts indicated by dotted white line. Scale bar, 10 µm.

Although the membrane of a spherical cell displays a rougher, more corrugated surface than a cell after adhesion and spreading (figure 4), the apparent area compressibility modulus 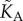 measured 5 min after cell seeding shows a value of 0.19 N m^−1^, essentially identical with a neat lipid bilayer in the absence of a buffering membrane reservoir. The area compressibility modulus of pure lipid bilayers and cell membranes is in the range of 0.1–1 N m^−1^, depending on their composition [18,13]. Cells with such high *K*_A_ values obviously possess a negligible amount of excess membrane area *A*_ex_. This confirms that a cell in its spherical state has no access to excess membrane at large strains, and therefore appears stiff. This is also supported by the large forces required to pull tethers from cells in the spherical state.

High tether forces suggest a stronger membrane binding to the underlying cortex. In order to provide additional evidence for our model, we labelled the F-actin in these spherical cells (see electronic supplementary material, figure S2*a*) displaying a uniform cortex. The blebs or protrusions occurring in the initial stage after adhering are therefore lined with actin filaments. This continuous and rigid actin cortex provides resistance to mechanical forces, and therefore protection against damage. Hence, non-adhering cells or cells just before adhesion show a high area compressibility modulus and high membrane tension (figure 3*a*).

### 3.5. First and second phase of cell spreading: adhesion results in tension loss

Within 10 min after seeding, cells adhere to the substrate by the formation of an initial adhesion zone [13]. Exactly at this time, we detect a relaxation in tension and simultaneously a sudden increase in the available membrane area. This is reflected by a lower area compressibility module 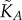 compared with the value of a lipid bilayer. The decrease in 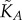 corresponds to an increase in excess membrane area by a factor of 12 (equation (3.4)). Additionally, we observe a disruption of the F-actin layer in the basal area of an adhering cell (see electronic supplementary material, figure S2, arrow) accompanied by a reduction in membrane tension (figure 3*b*,*c*), confirming a weakening of F-actin that supports the membrane. This temporary release in tension enables the cell to spread on the substrate by forming membrane protrusions, also visible in light microscopy (figure 1*d*) and fluorescence micrographs (figure 4*b*) as large spherical extensions (blebs) in the lower part of the cell. The breakdown of the F-actin cortex is followed by a bulk flow of cytoplasm, causing blebs to protrude, which allows spreading on the surface [16]. The nucleation of a membrane bleb is provoked by a local weakening of the cortical complex and promoted by the internal pressure of the cell generated by the contractile actomyosin cortex [13,20,27]. The drop in tension causes the change from a static and rigid to a more dynamic actin cortex that allows spreading by drawing additional membrane from reservoirs stored in protrusions, invaginations or internal membrane structures [23,28]. Ren and co-workers [29] observed a decrease in Rho activity when suspended cells adhere to a matrix. Rho regulates the formation of actin stress fibres [30] and integrin signalling [31]. Upon inhibition of Rho, the cortical F-actin network is partly disrupted, thereby allowing integrins to be clustered, which are otherwise dispersed over the cell surface in a suspended cell [32,33]. This allows the cell to initially adhere and finally to spread. We therefore attribute the decrease in tension and increase in excess membrane area to a breakdown of the cortical F-actin in the basal part of the cell, causing membrane material to protrude at these defects, which eventually form the initial adhesion zone.

### 3.6. Inhibition of adhesion abolishes tension drop

The adhesion of a spherical cell to the substrate leads to a release of tension and recruitment of excess membrane area. If this hypothesis is correct, then the inhibition of adhesion should prevent a change of mechanical parameters that mirror this process. Hence, we investigated the behaviour of an MDCK II cell in suspension by coating the substrate with bovine serum albumin (BSA), therefore abolishing cellular adhesion to a large extent [34].

**Figure 5.**
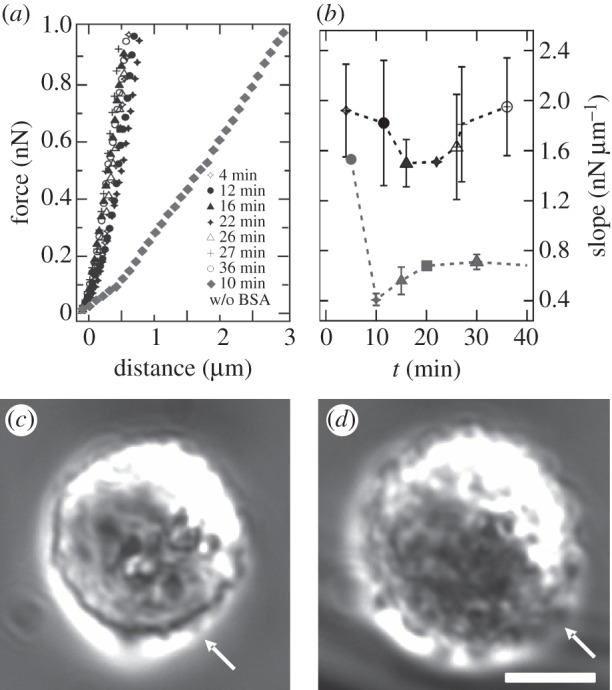
Inhibition of cell adhesion through passivation of the surface. (*a*) Representative force–distance curves taken over time on a non-spreading MDCK II cell in contact with a BSA-coated surface. Data are obtained from three independent cells. Grey diamonds represent FC recorded on a non-perturbed cell after 10 min of cell seeding, whereas all other curves are force curves taken on a cell on a BSA-coated surface. (*b*) Corresponding slope of FC as a function of time (symbols as in (*a*)). For comparison, slope of a non-perturbed adhering and spreading cell is plotted (grey). (*c*,*d*) Phase contrast micrographs of an MDCK II cell in suspension loosely attached to the BSA-coated surface. Surface of the Petri dish is coated with BSA to inhibit cell adhesion. Cells display membrane protrusions, indicated by white arrows. Scale bar, 10 µm.

While challenging the cells mechanically (figure 5*a*,*b*), no specific cellular adhesion to the substrate is seen. In addition, the barely adhering cells display many protrusions emphasizing that they are arrested in the state of initial adhesion (figure 5*c*,*d*). Figure 5*a* shows FCs taken continuously on a non-adhering spherical MDCK II cell. Most importantly, no change in the slope of recorded FCs over a time period of more than 30 min is seen, and the indentation depth does not exceed 1 µm. Comparing these data with the slope of an FC taken 10 min after cell seeding on an untreated substrate (grey diamonds in figure 5*a*), it becomes evident that neither adhesion nor spreading of spherical cells occurs on a BSA-coated substrate. While the slope of the force curves recorded on cells seeded on untreated surfaces drops in the first 10 min (figure 5*b*, grey), no change in slope or morphology is found for cells on BSA-coated surface. In terms of mechanical parameters, the cells on BSA-coated surfaces are stiff spheres with no excess area (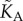 = (0.58±0.34) mN m^−1^) and dominated by cortical tension (*T*_c_ = (0.33±0.16) mN m^−1^), whereas membrane tension (*T*_t_ = (0.07±0.03) mN m^−1^) is low. In contrast to adhering cells displaying the same morphology in the early stage of adhesion that also possess a large 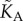, cortical tension is already substantially reduced. We attribute this to an early stage of mechanotransduction, occurring only on adhesive substrates in which tension release is important to allow further adhesion and spreading of the cell. This supports our picture that the adhesion process is accompanied by a characteristic change in tension and surface area, marked by an initial drop in tension followed by slow restoration of the initial tension.

We also performed chemical perturbation experiments on spreading cells by decreasing membrane tension with deoxycholate [13], destroying the actin cortex by administration of cytochalasin D and interfering with surface area regulation by using dynasore [35]. Interesting, all treatments led to a substantial perturbation of the spreading behaviour. Figure 3 (blue dots) shows the impact of cytochalasin D on spreading dynamics and mechanics. As expected, spreading and adhesion are hampered considerably, if the F-actin is disassembled. All mechanical parameters display a strong reduction, particularly those associated with an intact cytoskeleton. Cortical tension *T*_c_ is close to zero and membrane tension declines—the contribution from *γ*_ad_ is obviously lost. The value for the apparent area compressibility modulus after administration of cytochalasin D remains at low values (approx. 0.02 N m^−1^), which we explain with a large surface excess owing to loss of cytoskeleton integrity. The cells are not able to regulate the membrane surface area without an intact F-actin cytoskeleton, which is also observable in figure 2*f*, where abnormal membrane outgrowths are visible.

Membrane tension can be decreased by addition of detergents, organic solvents or local anaesthetics. We have investigated cell adhesion and spreading after administration of deoxycholate to reduce membrane tension (see electronic supplementary material, figure S3). Additionally, we observe neither adhesion nor spreading over a time period of more than 1 h. Indentation experiments on suspended cells treated with deoxycholate display an increase in indentation depth (approx. 2 µm), indicating softening due to decrease of membrane tension (see electronic supplementary material, figure S3*a*, grey). An appropriate geometrical description of the cellular shape, which is essential for applying a mechanical model, was not feasible for the blebbing cells. Therefore, we pulled membrane tethers to find a decrease in membrane tension *T*_t_ = (0.03 ± 0.01) mN m^−1^ compared with untreated cells (*T*_t_ ∼ 0.2 ± 0.01 mN m^−1^). This is in accordance with findings of Raucher and Sheetz [13], who treated NIH 3T3 fibroblasts with deoxycholate. The authors report an increase in the rate of lamellipodial extension and a decrease of tether forces by a factor of 2.

In contrast, suspended MDCK II cells treated with dynasore display no blebs, and the cell surface appears smooth (see electronic supplementary material, figure S4*c*). Because dynasore inhibits dynamin-dependent endocytosis, this treatment should strongly interfere with surface area regulation of the cells. Perturbation of surface area regulation should have a negative impact on the adhesion and spreading process. Indeed, we observe a slowing down of the spreading rate. First, adhesion to the substrate is detectable only after more than 1 h (see electronic supplementary material, figure S4*d*). Moreover, the apparent area compressibility modulus remains almost constant (approx. 0.2 N m^−1^; electronic supplementary material, figure S4*a*), which is indicative of a failing of surface area regulation due to the administration of dynasore. Simultaneously, neither cortical nor membrane tension (*T*_0_ ∼ 0.3 mN m^−1^, *T*_t_ ∼ 0.1 mN m^−1^) changes over time. In summary, cells treated with dynasore show a perturbed area regulation and stay arrested in an initial stage with constant mechanical parameters.

Taken together, we observe in all three experiments (addition of dynasore, deoxycholate, passivation with BSA) that the initial drop in tension and availability of excess membrane is a prerequisite for unperturbed adhesion and spreading of MDCK II cells.

### 3.7. Third phase of cell spreading

After adhesion and subsequent formation of membrane protrusions to enable spreading, the substrate adhesion zone of the cell increases. At the same time, membrane protrusions, invaginations and blebs are smoothed out. Figure 4 shows membrane staining of MDCK II cells during the adhesion and spreading process. Comparing the membrane roughness of just adhered cell with a fully spread one reveals a reduction of membrane protrusions after spreading (figure 4*a*–*c*). This is in agreement with a study of Norman *et al*., who describe that blebs disappear 30–40 min after seeding of cells [8]. While increasing the contact zone with the substrate by smoothing out membrane reservoirs, the apparent area compressibility modulus 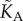 increases accordingly, reaching an almost constant modulus of 0.05 N m^−1^, which corresponds to a membrane folding factor of only 2–5 (figure 3*a*). Simultaneously, tension *T*_0_ measured by indentation, as well as tether pulling, shows a recovery to about 0.37 mN m^−1^ (figure 3*b*,*c*), mainly governed by cortical tension (figure 3*d*). At this time, first stress fibres become visible, indicative of fully established adhesion to the substrate (see electronic supplementary material, figure S2).

## 4. Conclusions

By combining our results from mechanical probing and fluorescence microscopy, we propose a mechanical model for the adhesion and spreading of a suspended MDCK II cell that captures the various phases from initial contact to a firmly adhered cell.

**Figure 6.**
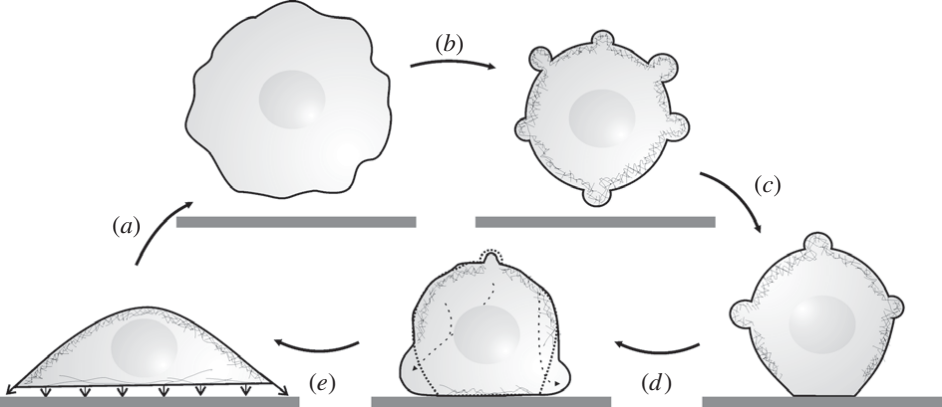
Illustration of the key events in cellular adhesion and spreading deduced from mechanical experiments as a function of time. (*a*) Detachment of an adherent cell due to trypsinization. (*b*) Freshly detached cells restore tension by creating actin-coated protrusions that consume excess membrane to maintain tension. (*c*) Owing to adhesion to a surface, the actin cortex partially opens close to the substrate, allowing (*d*) a bulk flow of cytoplasma to cause blebs as an onset of spreading. (*e*) In the last step, cells start to flatten and the cell's surface becomes smoother while stress fibres form.

Upon initial contact with a surface, a suspended cell shows a decrease of membrane tension, causing the visible formation of membrane protrusions (figure 6*a*). Tension is subsequently restored (figure 3*b*–*d*) by enclosing excess membrane into these actin-lined protrusions, reflected in a high area compressibility modulus close to that found for inextensible lipid bilayers mirroring absence of membrane reservoirs (figures 3*a* and 6*b*).

Adhesion to the substrate leads to a partial dissolution of the cortical actin and drop of tension, along with a marked increase in the excess membrane area (factor of 12; figure 6*c*). This is essential for the cell to spread by creating membrane protrusions originating from the basal part of the cell (figure 6*d*) [36]. Therefore, we postulate that cells that are able to establish mechanical contact to the substrate by molecular recognition events such as those between integrins and ECM [23] actively release tension of the cortex and membrane to permit recruitment of excess area stored in the membrane wrinkles and folds for further spreading. In the course of spreading, membrane tension increases again by smoothing out existing membrane reservoirs. After occurrence of first stress, fibres’ cortical and membrane tension as well as surface area maintain a constant level, indicating full adhesion of the cell to the substrate. Perturbation of cytoskeleton integrity, membrane tension and surface area regulation immediately arrests the cell in its initially spherical state, abolishing spreading on the substrate. In conclusion, time-resolved mechanical probing of cellular elasticity of the spreading process reveals that cell spreading requires a transient release in tension to permit formation of membrane protrusions that, in turn, allows the cell to spread. It is conceivable that a similar mechanism is responsible for the dynamic remodelling of the cytoskeleton during cell migration.

## 5. Material and methods

### 5.1. Cell culture

MDCK II cells, obtained from the Health Protection Agency, Salisbury, UK, were maintained in minimal essential medium with Earle's salts and 2.2 g l^−1^ NaHCO_3_ (Biochrom, Berlin, Germany) supplemented with 4 mM l-glutamine, penicillin–streptomycin and 10% FCS at 37°C in a 5% CO_2_-humidified incubator. Confluent cells were released with trypsin/EDTA (0.5%/0.2%; Biochrom) and subcultured weekly. For spreading studies, cells were trypsinized for 5 min and used within 30 min after detachment.

### 5.2. Perturbation experiments

We disrupted the actin cortex by exposing the suspended cells to cytochalasin D (10 μM; Sigma-Aldrich, Steinheim, Germany) for 15 min before seeding cells onto the Petri dish. To decrease membrane tension, we treated suspended cells with 0.4 mM deoxycholate (Sigma-Aldrich) for 30 min before cell seeding. We abolished endocytosis of suspended MDCK II cells by blocking dynamin with dynasore hydrate (100 μM; Sigma-Aldrich) 60 min before cell seeding.

### 5.3. Cell labelling

Cells in suspension were seeded on Ibidi Petri dishes (Ibidi, Martinsried, Germany) and incubated for different time intervals (5, 15, 30 min). Cells were fixed with 4% paraformaldehyde for 30 min. F-actin labelling was carried with Alexa 488-phalloidin (Invitrogen, Darmstadt, Germany; diluted as recommended by the manufacturer, 5 µl methanolic stock solution with 200 µl dilution buffer for each coverslip). The dye was diluted in dilution buffer (1% (w/v) BSA, 0.3% (w/v) Triton-X 100 in phosphate-buffered saline, PBS), which also permeabilizes the cells. Membrane labelling of living cells was carried out by incubating cells for 5 min with CellMask deep red plasma membrane staining solution (5 µg ml^−1^; Life Technology, Frankfurt, Germany) at 37°C. Cells were fixed after staining as described above. Fluorescence imaging was carried out with fixed cell using an upright confocal laser scanning microscope (CLSM; LSM710, Zeiss, Jena, Germany) equipped with a water immersion objective with 63× magnification.

### 5.4. Phase contrast imaging during spreading

The spreading process was observed on living MDCK II cells during the mechanical investigation on an Olympus IX 81 inverted light microscope equipped with long-distance phase contrast objective (40× magnification).

### 5.5. Atomic force microscopy for elasticity measurements

Force curves were recorded with a nanowizard II AFM (JPK Instruments AG, Berlin, Germany) mounted on an Olympus IX 81 inverted light microscope. MDCK II cells seeded on Ibidi Petri dishes were mounted in a Petri dish heater (JPK Instruments) set to 37°C with HEPES-buffered culture medium. Force curves were taken in the middle of a single cell with a cantilever having a nominal spring constant of 10 mN m^−1^ (MLCT, Bruker AFM, Proes, Camarillo, CA, USA). The shape of the cell parametrized by the base radius and the contact angle was computed at each indentation depth, which allowed us to fit 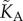 and *T*_0_ to experimental force indentation models [15]. A home-made Matlab script using a simplex algorithm was used for this purpose. All measurements were recorded with a set point of 1 nN normal force and tip velocities of 2 µm s^−1^. The opening angle *α* of the tip indenter was 17.5°, and the radius of the cell *R*_c_ was set to 11 µm. Small variations in *R*_c_ did not significantly affect the outcome of the fitting procedure.

### 5.6. Atomic force microscopy for probing tether pulling

Prior to tether pulling, cantilevers (MLCT, Bruker AFM, Proes) were plasma cleaned for 30 s (Argon) and incubated with 2.5 mg ml^−1^ concanavalin A (Sigma-Aldrich) in PBS for 1.5 h to establish a strong contact to the apical surface. Approach and retrace velocities were both set to 2 µm s^−1^. Tether forces were converted into tension values *T*_t_ using equation (3.5).

### 5.7. BSA coating of the substrate to avoid adhesion

For the inhibition of adhesion of the suspended cells to the substrate by saturating unspecific binding sites, we functionalized Petri dishes with BSA. Therefore, we incubated the Petri dish with 5% BSA in PBS for 20 min at 37°C. After mild rinsing to remove excess BSA in solution, cell suspension was added to the substrate.

## Supplementary Material

Additional figures S1-S4
