# Supplementary material for: Mechanics of spreading cells probed by atomic force microscopy
Source: Open Biol. 2013 Jul;3(7):130084. doi: 10.1098/rsob.130084 (PMC3728925; doi:10.1098/rsob.130084)
Supplement: Additional figures S1-S4 [file rsob130084-s1.pdf]

Supplementary Information

Mechanics of spreading cells probed by AFM

Anna Pietuch and Andreas Janshoff*

Institute of Physical Chemistry, Georg-August-University Goettingen, Tammannstrasse 6, 37077 Goettingen, Germany.

* correspondence to: Prof. Dr. Andreas Janshoff, Tel. +49 551 39 10663, Fax. +49 551 39 14411, e-mail address: ajansho@gwdg.de.

Figure S1:

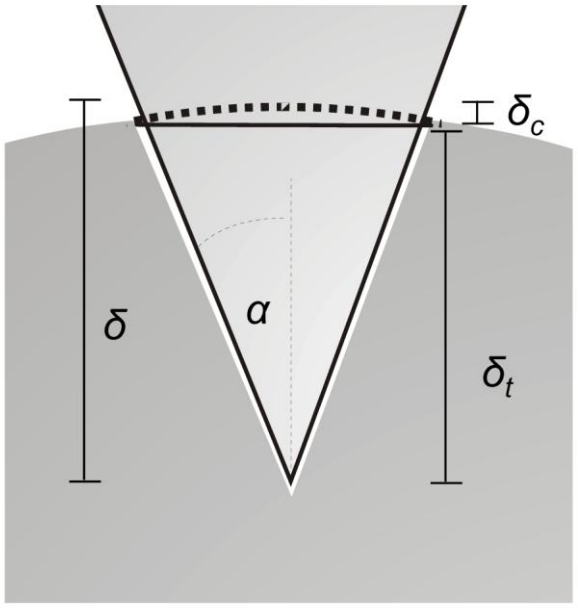

Figure S2:

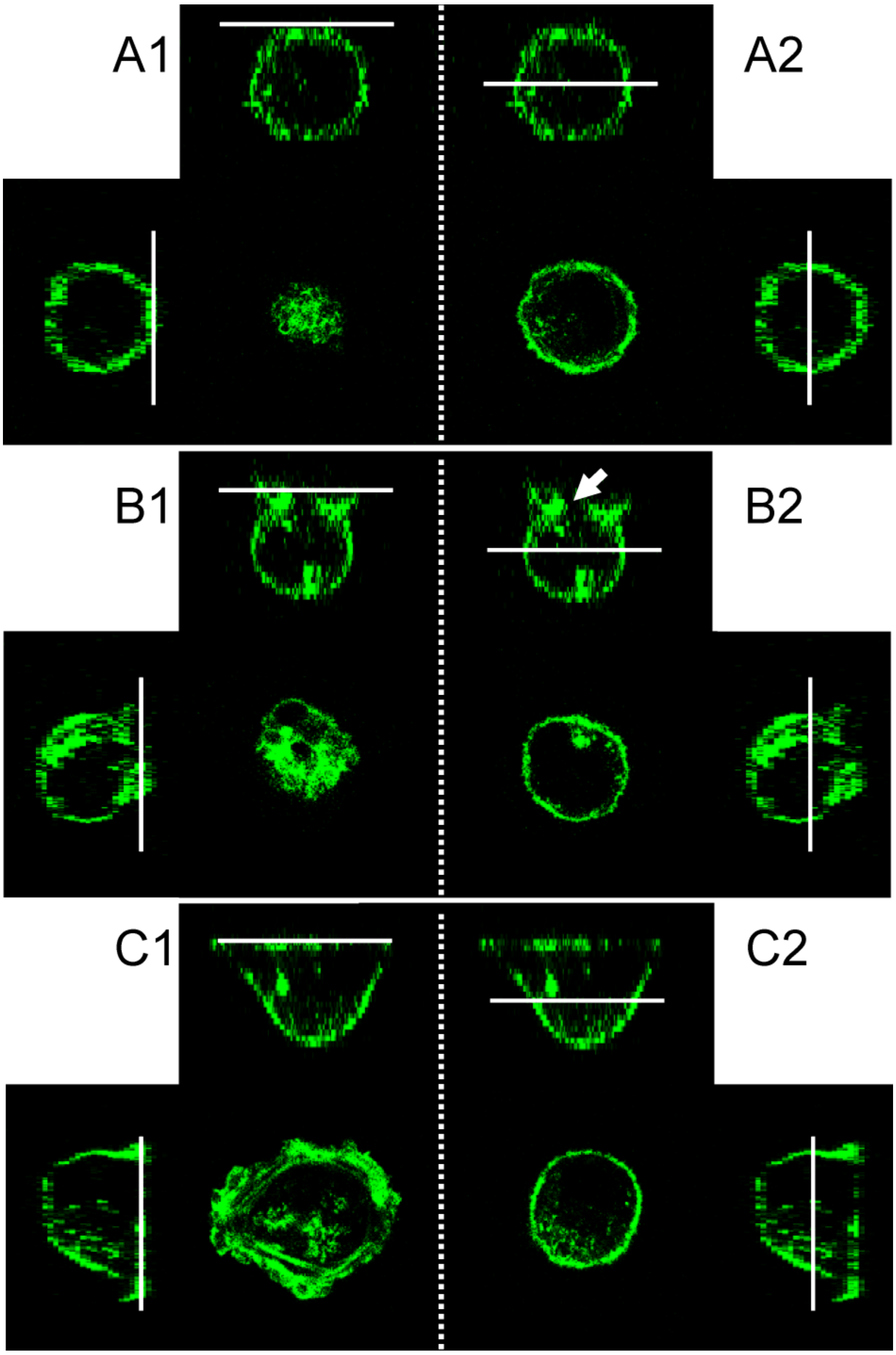

Figure S3:

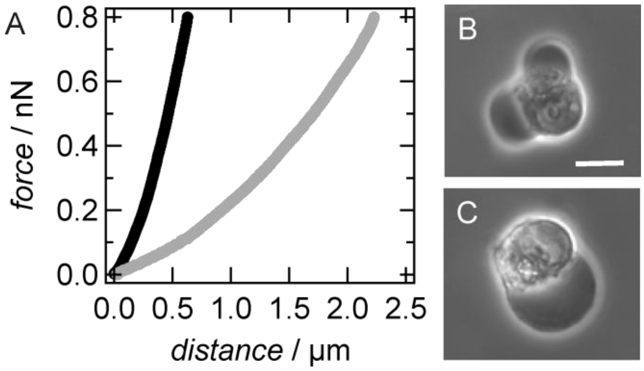

Figure S4:

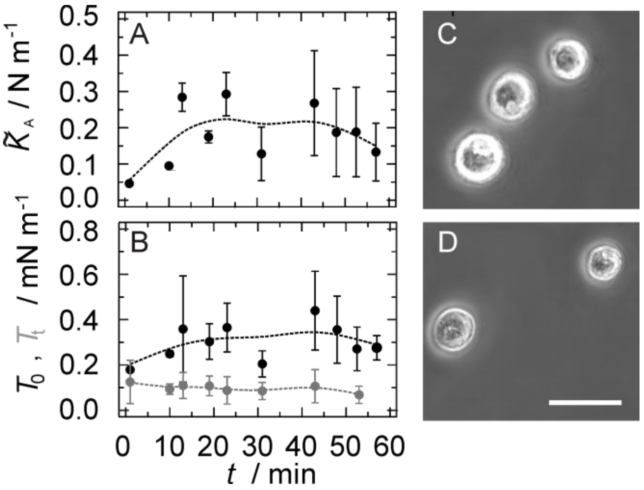

A
B
C
D
$\tilde{K}_A$ / N m^{-1}
T_0 , T_t / mN m^{-1}
t / min
0.0
0.1
0.2
0.3
0.4
0.5
0.0
0.2
0.4
0.6
0.8
0
10
20
30
40
50
60
